# Listening to music as a stress management tool

**DOI:** 10.1192/j.eurpsy.2021.1621

**Published:** 2021-08-13

**Authors:** F. Lata, I. Kourtesis

**Affiliations:** Department of Adult Psychiatry, Psychiatric Hospital of Athens “Dafni”, ATHENS, Greece

**Keywords:** music listening, stress management

## Abstract

**Introduction:**

The impact of listening to music on the stress response system has received increased attention lately.

**Objectives:**

An update of the relative research.

**Methods:**

Literature review.

**Results:**

Listening to music seems to reduce stress by influencing both the hypothalamic-pituitary-adrenal axis and the autonomous nervous system. Most studies, mainly conducted in healthy adults, show a decrease both in cortisol levels (a well-known stress-biomarker) and in sympathetic activity (reduction in heart rate frequency and blood pressure). Compositional elements of music such as melody, rhythm, tonality and frequency seem to influence individual relaxation responses. Most studies used classical music, nonetheless, the abovementioned effects were noticed irrespective of music genre. High-frequency music seems to have a greater role in stress-relief: 528 Hz frequency music apparently lowers cortisol and increases oxytocin (a modulator of stress response and social bonding) levels. A decrease in perceived anxiety and the induction of a positive mood state -particularly when relaxation was affirmed as the purpose of music listening- has been noticed using appropriate scales. Regarding particular settings, perioperative music may attenuate the neuroendocrine stress response caused by the surgery procedure. Music interventions in obstetric patients as well as in cardiovascular and cancer patients have led to similar findings. Finally, music appears to beneficially affect stress among patients with PSTD, fibromyalgia and depression.

**Conclusions:**

The stress-relieving effect of music listening seems promising in clinical settings. The heterogeneity of the studies’ sample and the “administration” of different music intervention “schemes” are among the main limitations of the current research.

**Disclosure:**

No significant relationships.

